# Chromosomal and reproductive features of some Oriental and Australasian scale insects (Homoptera, Coccinea)

**DOI:** 10.3897/CompCytogen.v14i3.53367

**Published:** 2020-07-20

**Authors:** Ilya A. Gavrilov-Zimin

**Affiliations:** 1 Zoological Institute, Russian Academy of Sciences, Universitetskaya nab. 1, St. Petersburg, 199034, Russia Zoological Institute, Russian Academy of Sciences St. Petersburg Russia

**Keywords:** scale insects, giant scales, mealybugs, soft scales, felt scales, chromosome number, karyotype, genetic system, reproductive system

## Abstract

Fourteen species of scale insects from the families Margarodidae s.l., Pseudococcidae, Eriococcidae, and Coccidae were investigated for the first time in respect to karyotypes, genetic systems, modes of reproduction and general anatomy of the female reproductive system. One of the studied species, *Steatococcus samaraius* Morrison, 1927, showed hermaphroditic reproduction of the female-like specimens, the other species demonstrated bisexual reproduction with a peculiar “Lecanoid” heterochromatinization of the paternal set of chromosomes in male embryos or thelytocous parthenogenesis. *Antonina parazonata* Williams, 2004 and *Saccharolecanium krugeri* (Zehntner, 1897) are recorded here for the first time from Thailand, *Antonina vietnamensis* Williams, 2004 and *Geococcus satellitum* Williams, 2004 – for the first time from Laos.

## Introduction

The present paper continues a series of the author’s publications on the cytogenetics and reproductive biology of scale insects from different regions of the world (Gavrilov 2004, 2007, Gavrilov and Kuznetsova 2007, Gavrilov and Trapeznikova 2007, 2008, 2010, Gavrilov-Zimin 2011, 2012, 2016, 2017, 2018a, b, Gavrilov-Zimin et al. 2015). Here, 14 previously unstudied species from 13 genera of the families Margarodidae s.l., Pseudococcidae, Eriococcidae, and Coccidae are considered in respect of their karyotypes, genetic systems, modes of reproduction, and general anatomy of the female reproductive system. Unusual aberrant genetic systems of scale insects have been reviewed several times previously (e.g., Hughes-Schrader 1948, Nur 1980, Gavrilov 2007, Gavrilov-Zimin et al. 2015) and will not be discussed here. General evolutionary aspects of scale insect reproductive biology and ontogenesis were analyzed in a special monograph (Gavrilov-Zimin 2018a), that can also be used by readers for the clarifying of the terminology and the higher-level taxa system, explored below.

General anatomic types of the female reproductive system in the scale insects were previously reviewed by De Marzo et al. (1990) basing on a few, mainly European species. However, subsequent studies (for example, Gavrilov and Trapeznikova 2007, Gavrilov-Zimin 2012, 2018a), including the present work, support the view of the mentioned authors (l.c.) that the main types of the reproductive system are characteristic of the higher taxa of scale insects (families, subfamilies, tribes).

## Material and methods

Material was collected by the author in different years in Thailand, Laos, Malaysia and Indonesia (Sulawesi, Bali, New Guinea). The detailed collecting data are provided below for each species. All numbers with the letter “K” mean unique collecting numbers for both acetoethanol material and Canada balsam slides. All material is deposited at the Zoological Institute, Russian Academy of Sciences (ZIN RAS), St. Petersburg, Russia.

Both the method for the preparation of permanent morphological slides mounted with Canada balsam and the method of squashing the embryonic cells in lactoacetic orcein for chromosome studies were reported, for example, by Danzig and Gavrilov-Zimin (2014).

All figures and photos, excluding the colour ones, were prepared by the author. The colour photos were prepared by the author with a kind help of D.A. Gapon.

## Results and discussion

### Family Margarodidae s.l.

#### Steatococcus samaraius

Taxon classificationAnimaliaHemipteraMargarodidae

Morrison, 1927

15D6DA82-30BA-5345-A134-6EBD0121B8D2

Figs 1a , 2

##### Material.

K 922, Indonesia, Sulawesi, vicinity of Kendari, on twigs of undetermined bush, 10.XI.2011, I.A. Gavrilov-Zimin. K 1071, Malaysia, Borneo, Damai Peninsula, on inflorescences of palm tree (probably *Areca catechu* Linnaeus, 1753), 14.I.2013, I.A. Gavrilov-Zimin.

**Figure 1.**
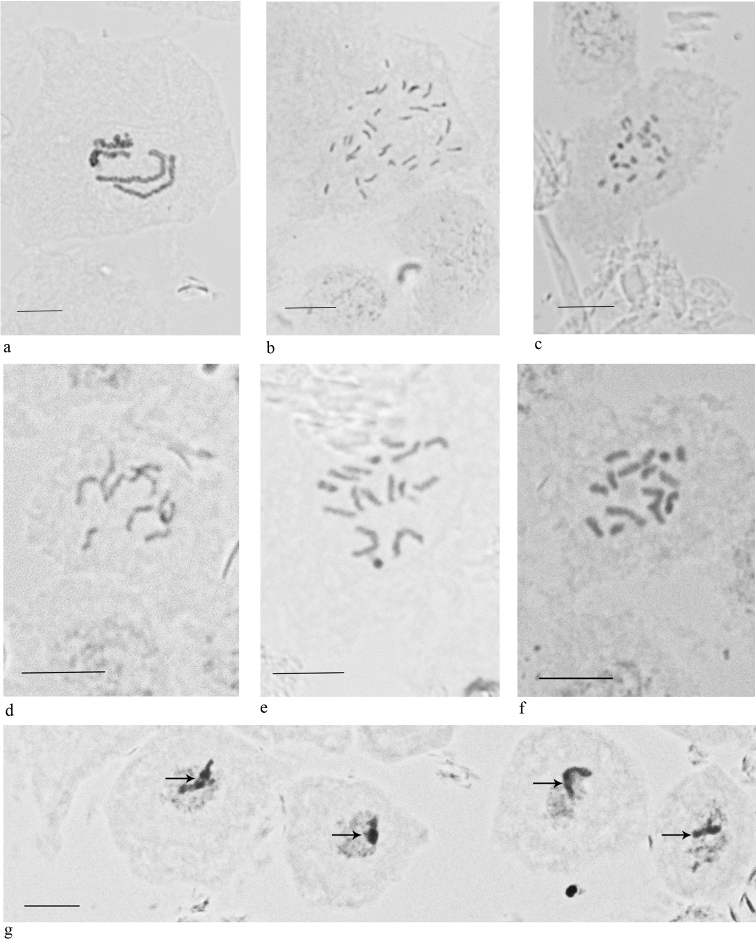
Embryonic cells and chromosomes of the studied species (Margarodidae, Pseudococcidae, Eriococcidae). **a***Steatococcus samaraius* (2n = 4) **b***Antonina parazonata* (2n = 30) **c***A. vietnamensis* (2n = 20) **d***Mollicoccus guadalcanalanus* (2n = 10) **e**Acanthococcus prope onukii (2n = 16) **f, g***Gossypariella siamensis* (2n = 16). **g** Shows interphase cells of male embryos with a Lecanoid heterochromatinization of the paternal set of chromosomes (arrowed in each cell). Scale bars: 10 µm.

##### New data.

**Figure 2.**
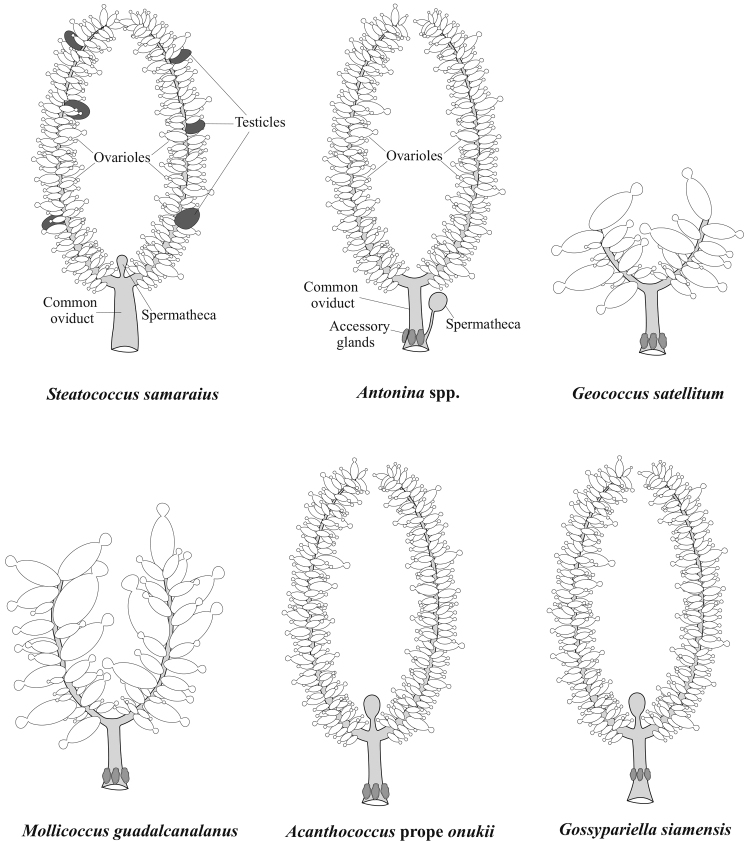
General anatomy of the female (or female-like hermaphrodite in *Steatococcus samaraius*) reproductive system in the studied species (Margarodidae, Pseudococcidae, Eriococcidae).

2n = 4; hermaphroditism: the studied female-like ultimolarvae contain sperm bundles in the ovo-testicles. Early stages of embryogenesis (before anatrepsis) occur inside of ovary; then the eggs are laid in the marsupium, where the embryogenesis ends. Hermaphroditic reproductive system is generally similar to the usual female system in bisexual species of scale insects, but contains testicular parts, located between numerous ovarioles (Fig. 2).

##### Comments.

Hermaphroditism is an exceptionally rare phenomenon in Insecta (see, for example, Royer 1975). Up to now hermaphroditic species are known for sure only in the scale insect tribe Iceryini (Margarodidae: Monophlebinae) (Hughes-Schrader 1948, Gavrilov 2007, Gavrilov-Zimin 2018a). Previously, the presence of ovo-testicles in female-like diploid insects has been shown for *Icerya bimaculata* De Lotto, 1959 (Hughes-Shrader 1963), *I. multicicatrices* (Kondo & Unruh, 2009) (Gavrilov-Zimin 2018a: 27, 190) and *I. purchasi* Maskell, 1879 (Schrader and Hughes-Schrader 1926, Royer 1975). Closely related genus *Steatococcus* Ferris, 1921 (18 species), which differs from *Icerya* Signoret, 1876 (45 species) by the presence of peculiar marsupium, was previously almost unstudied in respect of cytogenetics and reproductive biology, excluding the only American species, *S. tuberculatus* Morrison, 1941. This species was investigated by Hughes-Schrader and Ris (1941) who found that it had 2n = 4 and reproduced bisexually with the appearance of haploid males via facultative parthenogenesis. Here, another species of the genus, *S. samaraius*, a widely distributed Oriental and Australasian pest, was studied and the same chromosome number, 2n = 4, was discovered. However, males were totally absent in any populations of *S. samaraius*, inspected by me in the different countries of the Oriental region and seem to have never been reported in the literature. The preparation of the mature females and larvae expectedly revealed a hermaphroditic condition of the reproductive system of *S. samaraius*. Such a combination of hermaphroditism and haplo-diploidy in closely related species of one genus and even in different populations of the species (as is the case of *Icerya purchasi*; Schrader & Hughes-Schrader 1926) is a peculiar feature of the tribe Iceryini (Hughes-Shrader, 1963, Gavrilov-Zimin 2018a). Some authors (Unruh and Gullan 2007) do not consider *Steatococcus* as a separate genus and place its species either in the genus *Icerya* or in another related genus *Crypticerya* Cockerell, 1895. However, such approach leads to the total overlapping of the generic diagnostic characters and to the practical impossibility of assigning newly described species to a certain genus (see Gavrilov-Zimin 2018a: 174, 184, Gavrilov-Zimin and Stekolshikov 2018).

### Family Pseudococcidae

#### Antonina parazonata

Taxon classificationAnimaliaHemipteraPseudococcidae

Williams, 2004

87FA63D0-4F8A-5EA8-B8B2-F6D85ECB101C

Figs 1b , 2

##### Material.

K 1533, Thailand, Pai, the road to Mae Yen Luang waterfalls, on twigs of bamboo, 13.XI.2019, I.A. Gavrilov-Zimin.

##### New data.

2n = 30; bisexual reproduction with a Lecanoid heterochromatinization of paternal chromosomes in male embryos; complete ovoviviparity. Female reproductive system is similar to that of other studied mealybugs, i.e. with numerous ovarioles located on the paired oviducts, accessory glands attached to the proximal part of the common oviduct, and a spermatheca located at the same place as accessory glands (Fig. 2)

##### Comments.

Special study of cytogenetics and reproductive biology of the genus *Antonina* Signoret, 1875 and other “legless mealybugs” was done recently (Gavrilov-Zimin 2016). Nine species from 3 genera of legless mealybugs were considered in that paper based on original and literature data and a significant variation of chromosome number was shown: 2n = 10, 12, 16, 20, 22+ Bs, 24, 24 + Bs, and 30. *Antonina parazonata*, studied here showed 2n = 30 as a species from the related monotypic genus *Komodesia* Gavrilov, 2016, namely, *Komodesia circuliplurima* Gavrilov, 2016. For the genus *Antonina*, such a high chromosome number was revealed for the first time.

*A. parazonata* was previously known from the type localities in India only. It is the first record of this species for Thailand.

#### Antonina vietnamensis

Taxon classificationAnimaliaHemipteraPseudococcidae

Antonina

Williams, 2004

98E09AC4-1FD3-5EFF-AEEA-7249A5889F8E

Figs 1c , 2

##### Material.

K 1380, Laos, Pak Beng, on twigs of bamboo, 13.VI.2017, I.A. Gavrilov-Zimin.

##### New data.

2n=20; bisexual reproduction with a Lecanoid heterochromatinization in male embryos; complete ovoviviparity. Female reproductive system is the same type as in *A. parazonata* (Fig. 2).

##### Comments.

*Antonina vietnamensis* has the same chromosome number as a closely related Oriental species of the genus, *A. diversiglandulosa* Gavrilov, 2016.

*A. vietnamensis* was previously known from the type localities in Vietnam only. It is the first record of the species for Laos.

#### Geococcus satellitum

Taxon classificationAnimaliaHemipteraPseudococcidae

Williams, 2004

E3F9D0BD-C12A-54C6-8B56-0A498B26A5AE

Fig. 2

##### Material.

K 1382, Laos, Pak Beng, on roots of dicotyledonous herb, 13.VI.2017, I.A. Gavrilov-Zimin.

##### New data.

All studied embryos from 3 available females were unsuitable for chromosomal studies due to numerous yolk inclusions. Eggs are laid in loose ovisac at the stage of anatrepsis suggesting incomplete ovoviviparity. Female reproductive system is characterized by an extremely small number of ovarioles and the absence of a spermatheca (Fig. 2).

##### Comments.

Up to now, the genus *Geococcus* Green, 1902 (14 species) has not been studied in terms of cytogenetics and reproductive biology. This is the case with most other related genera of tribe Rhizoecini (or group of the genus *Rhizoecus* Künckel d’Herculais, 1878). Diploid chromosome numbers, 8, 10, and 12, are known only for 5 species of *Rhizoecus* (Danzig & Gavrilov-Zimin 2015: 428–429); all these species are characterized by a Lecanoid genetic system and bisexual reproduction.

*G. satellitum* was previously known from the type localities in China and Thailand only. This is the first record of this species for Laos.

#### Mollicoccus guadalcanalanus

Taxon classificationAnimaliaHemipteraPseudococcidae

Williams, 1960

9207C031-09E5-5FC5-A472-175F3CA0A509

Figs 1d , 2

##### Material.

K 917, Indonesia, New Guinea, Manokwari, forest near the airport, on leaves of undetermined dicotyledonous herb, 8.XI. 2011, I.A. Gavrilov-Zimin.

##### New data.

2n = 10; bisexual reproduction with a Lecanoid heterochromatinization in male embryos; eggs are laid in loose ovisac at stage of anatrepsis suggesting incomplete ovoviviparity. Female reproductive system is similar in general details to that of *Geococcus satellitum* (Fig. 2).

##### Comments.

These are the first cytogenetic and reproductive data for monotypic Australasian genus *Mollicoccus* Williams, 1960. The diploid number 10 is considered a modal chromosome number for the family Pseudococcidae as a whole (Nur 1980, Gavrilov 2007, Gavrilov-Zimin et al. 2015).

### Family Eriococcidae

#### Acanthococcus prope onukii

Taxon classificationAnimaliaHemipteraEriococcidae

(Kuwana, 1902)

794F6C47-6D9E-5E76-9897-227170178D23

Figs 1e , 2

##### Material.

K 1513, Thailand, Chiang Mai, slope of Doi Suthep Mt. near the University, on leaves of bamboo, 8.XI.2019, I.A. Gavrilov-Zimin.

##### New data.

2n = 16; bisexual reproduction with a Lecanoid heterochromatinization in male embryos. Eggs are laid in dense wax ovisac at the stage of anatrepsis, i.e. incomplete ovoviviparity is characteristic of the species. Female reproductive system consists of a spermatheca attached at the junction of the oviducts and accessory glands attached at the base of a common oviduct (Fig. 2).

##### Comments.

Only two species of the large genus *Acanthococcus* Signoret, 1875 have been previously studied cytogenetically, i.e. European *A. agropyri* (Borchsenius, 1949) and *A. insignis* (Newstead, 1891), both with 2n = 16 (Gavrilov 2004, 2007). [Nota bene! The studied specimens differ from a common *Acanthococcus onukii* (=*Anophococcus onukii*) in the conical setae with blunt apices. The generic name *Anophococcus* Balachowsky, 1954 is considered here as a synonym of *Acanthococcus* (synonymized by Danzig 1980: 205)].

#### Gossypariella siamensis

Taxon classificationAnimaliaHemipteraEriococcidae

(Takahashi, 1942)

FC64B7CD-4B5E-5352-A070-0785017FA285

Figs 1f–g , 2 , 5a

##### Material.

K 1521, Thailand, Chiang Mai, city street near the University, on branches and twigs of an undetermined dicotyledonous tree, probably *Ficus* sp., 9.XI.2019, I.A. Gavrilov-Zimin.

##### New data.

2n = 16; bisexual reproduction with a Lecanoid heterochromatinization in male embryos. Complete ovoviviparity. Female reproductive system is similar with that in the previous species, but accessory glands are located in the middle part of the common oviduct (Fig. 2).

##### Comments.

The genus *Gossypariella* Borchsenius, 1960 includes 4 species distributed in the Oriental region. *G. siamensis* is the first species of the genus studied cytogenetically.

### Family Coccidae

#### Coccus viridis

Taxon classificationAnimaliaHemipteraCoccidae

(Green, 1889)

C7E5B425-7483-5FCB-B95F-E643E84A9C09

Figs 3a , 4

##### Material.

K 939, Indonesia, Bali, mountain forest above Lake Buyan, about 1200 m altitude, on leaves of an undetermined tree, 13.XI. 2011, I.A. Gavrilov-Zimin.

**Figure 3.**
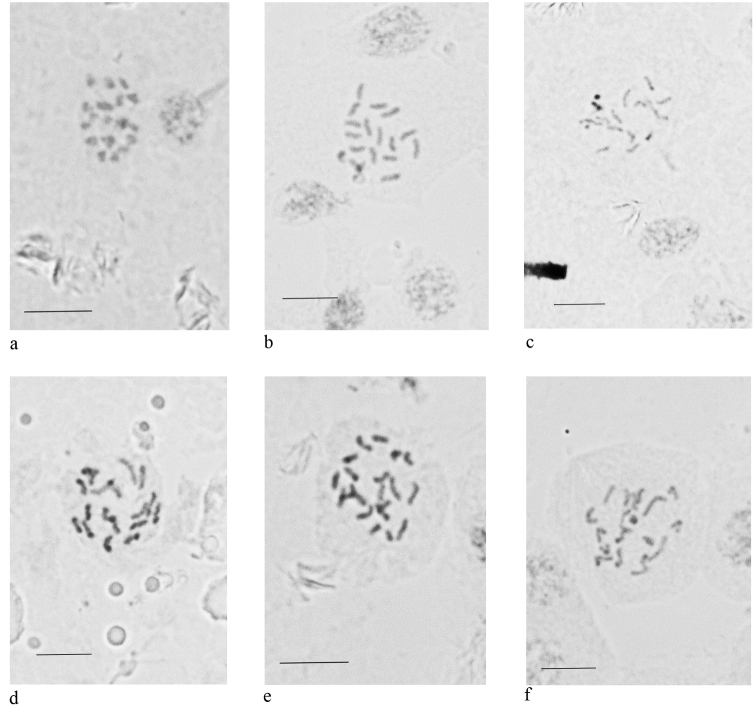
Embryonic cells and chromosomes of the studied species (Coccidae). **a***Coccus viridis* (2n = 18) **b***Discochiton expansum* (2n = 18) **c***Luzulaspis australis* (2n = 18) **d***Megalocryptes buteae* (2n = 18) **e***Megapulvinaria maxima* (2n = 20) **f***Saccharolecanium krugeri* (2n = 18). Scale bars: 10 µm.

##### New data.

**Figure 4.**
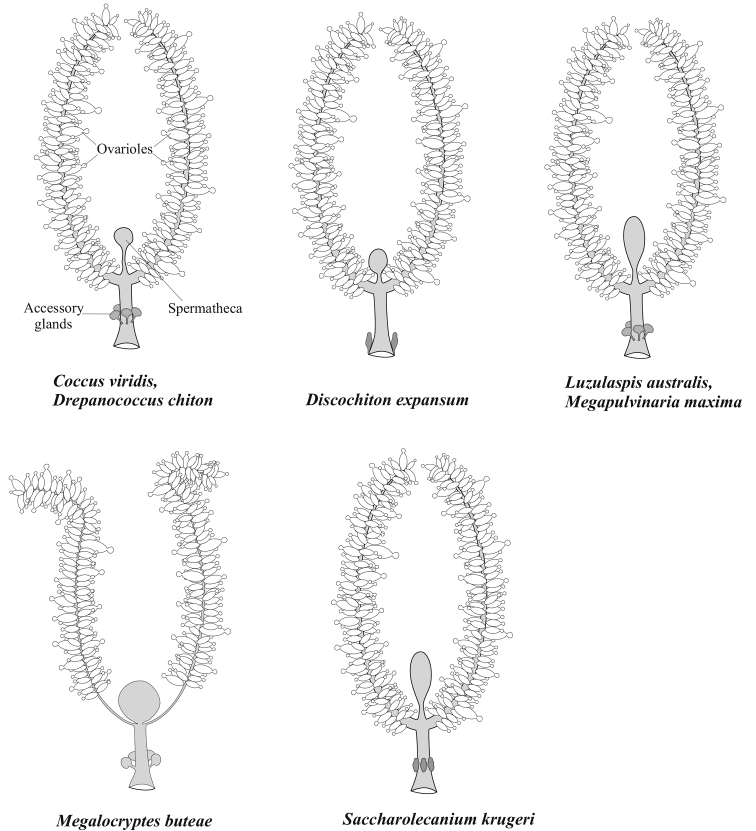
General anatomy of the female reproductive system in the studied species (Coccidae).

2n = 18; there is no heterochromatinization (and thus no Lecanoid system) in all 50 studied embryos from 3 females, no sperm in spermathecae and no males in the studied population; so, the thelytocous reproduction is characteristic of this species. Complete ovoviviparity. Female reproductive system is of the usual for the soft scales type (Fig. 4).

##### Comments.

The type species of the genus, *Coccus hesperidum* Linnaeus, 1758, shows 2n =14 and different variants of parthenogenesis (Thomsen 1927, 1929, Nur 1979), whereas two other studied species, *C. longulus* (Douglas, 1887) and *Coccus* sp., were reported by Moharana (1990) as having 2n=18, but without any comments on genetic system and reproductive peculiarities . All other (more than 110) species of the genus *Coccus* Linnaeus, 1758, are still unstudied cytogenetically.

**Figure 5.**
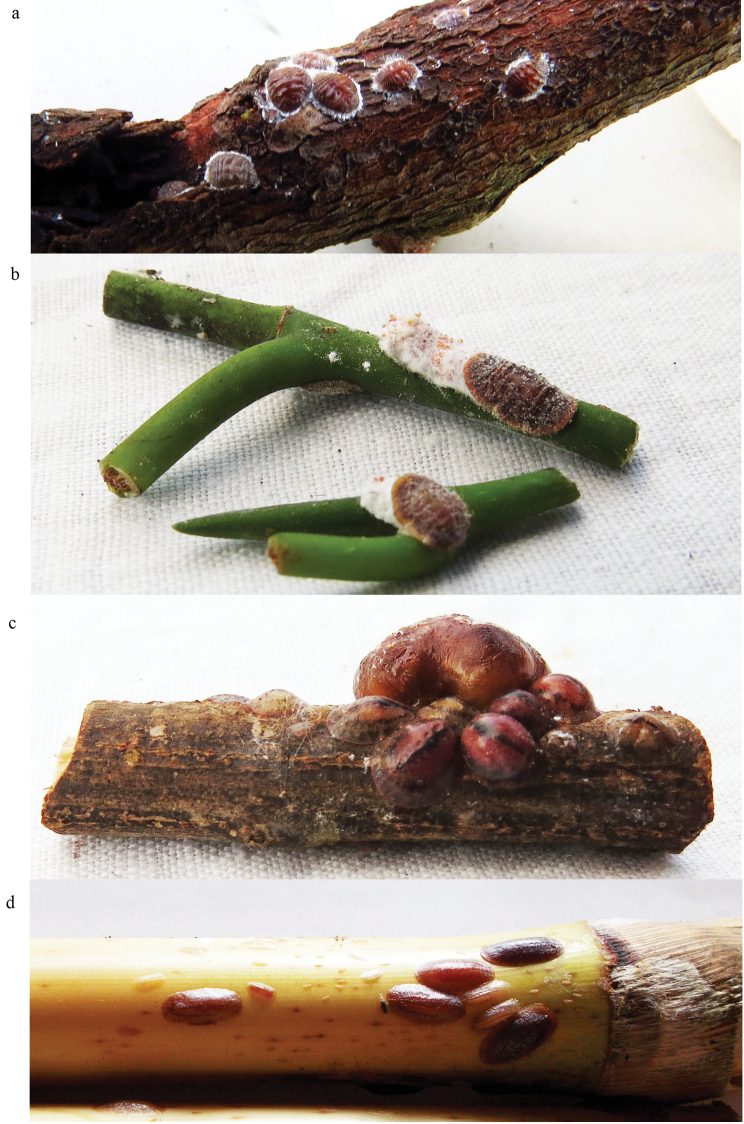
Females of some species on twigs of host plants. **a***Gossypariella siamensis***b***Megapulvinaria maxima***c***Megalocryptes buteae* (with a colony of *Kerria* sp. at the background) **d***Saccharolecanium krugeri*.

#### Discochiton expansum

Taxon classificationAnimaliaHemipteraCoccidae

(Green, 1896)

F47D985E-A5E1-56D0-94B5-4C9B6A024279

Figs 3b , 4

##### Material.

K 1121, Thailand, Malay Peninsula, Khao Lak, forest above the city, on leaves of an undetermined bush, 8.XI. 2013, I.A. Gavrilov-Zimin.

##### New data.

2n = 18; bisexual reproduction with a Lecanoid heterochromatinization in male embryos. Complete ovoviviparity. Female reproductive system has the usual structure, but accessory glands are poorly visible (Fig. 4).

##### Comments.

The recently erected genus *Discochiton* Hodgson & Williams, 2018 comprises 21 species, and *D. expansum* is the first species of the genus studied cytogenetically.

#### Drepanococcus chiton

Taxon classificationAnimaliaHemipteraCoccidae

(Green, 1909)

8E2A203D-8144-5117-8BE8-0B6645AA4177

Fig. 4

##### Material.

K 864, Indonesia, New Guinea, vicinity of Jayapura, Entrop, on stem of a dicotyledonous herb, 30.X. 2011, I.A. Gavrilov-Zimin.

##### New data.

There were no embryonic cells suitable for chromosomal analysis in the available material. The reproduction is bisexual with a Lecanoid heterochromatinization in male embryos. All studied females contained embryos at early stages of embryogenesis (up to anatrepsis). Female reproductive system has the usual structure (Fig. 4).

##### Comments.

The only other species of the genus, *D. cajani* (Maskell, 1891), was previously studied cytogenetically by Moharana (1990), who reported 2n = 18 with no other comments on the species.

#### Luzulaspis australis

Taxon classificationAnimaliaHemipteraCoccidae

(Maskell, 1894)

0B11C984-A5FB-5ABA-B1B5-B7E61DDCFB38

Figs 3c , 4

##### Material.

K 861, Indonesia, New Guinea, vicinity of Jayapura, Entrop, under leaf sheathes of a Poaceae grass, 30.X. 2011, I.A. Gavrilov-Zimin.

##### New data.

2n = 18; bisexual reproduction with a Lecanoid heterochromatinization in male embryos. The eggs are laid in a long wax ovisac at the stage of late anatrepsis; i.e. incomplete ovoviviparity is characteristic of the species. Female reproductive system has the usual structure (Fig. 4).

##### Comments.

The genus *Luzulaspis* Cockerell, 1902 comprises about 25 species, but only one of them, European *L. dactylis* Green, 1928, has been thus far studied cytogenetically (Gavrilov 2004). This species was found to have 2n = 18 and a bisexual reproduction with a Lecanoid heterochromatinization as well as presently studied Australasian *L. australis*.

#### Megalocryptes buteae

Taxon classificationAnimaliaHemipteraCoccidae

Takahashi, 1942

202BC752-919C-54FE-8CA9-774A534338AB

Figs 3d , 4 , 5c

##### Material.

K 1536, Thailand, Pai, on twigs of an undetermined dicotyledonous tree, 13.XI.2019, I.A. Gavrilov-Zimin.

##### New data.

2n = 18; there is no heterochromatinization in all 72 studied embryos from 3 females, no sperm in spermathecae and no males in the population suggesting thus thelytokous reproduction. Female reproductive system is distinguished by unusually long and thin lateral oviducts (Fig. 4). Cleavage divisions in the egg start just prior to oviposition.

##### Comments.

These are the first cytogenetic and reproductive data for the small Oriental genus *Megalocryptes* Takahashi, 1942 which comprises two species only.

#### Megapulvinaria maxima

Taxon classificationAnimaliaHemipteraCoccidae

(Green, 1904)

5F0B9BF4-187F-5D96-8B52-64F95BD937F9

Figs 3e , 4 , 5b

##### Material.

K 1531, Thailand, Pai, on leaves and twigs of an undetermined dicotyledonous tree, 13.XI.2019, I.A. Gavrilov-Zimin.

##### New data.

2n = 20; bisexual reproduction with a Lecanoid heterochromatinization in male embryos. Incomplete ovoviviparity: embryogenesis (until the late anatrepsis) partially occurs inside of the mother’s body. Female reproductive system has the usual structure (Fig. 4).

##### Comments.

Different European members of the tribe Pulvinariini have been previously studied cytogenetically (Gavrilov 2007, Gavrilov and Trapeznikova 2008). Four Oriental species from the genera *Chloropulvinaria* Borchsenius, 1952, *Pseudopulvinaria* Atkinson, 1889 and *Pulvinaria* Targioni Tozzetti, 1866 were studied by Moharana (1990), who reported chromosome numbers with no comments or details. *M. maxima* is the first species of the genus *Megapulvinaria* Yang, 1982 studied in terms of chromosome number; the kartotype 2n = 20 is found for the first time in the tribe Pulvinariini in general.

#### Saccharolecanium krugeri

Taxon classificationAnimaliaHemipteraCoccidae

(Zehntner, 1897)

25333FE7-C5B6-545B-A9EC-C492BBD389C5

Figs 3f , 4 , 5d

##### Material.

K 1368, Thailand, vicinity of Chiang Rai, forest above the Mae Fah Luang University, under the leaf sheathes of ?*Saccharum* sp., 8.VI.2017, I.A. Gavrilov-Zimin.

##### New data.

2n = 18; bisexual reproduction with a Lecanoid heterochromatinization in male embryos. Complete ovoviviparity. Female reproductive system has the usual structure (Fig. 4).

##### Comments.

These are the first cytogenetic and reproductive data for the small Oriental genus *Saccharolecanium* Williams, 1980, which comprises two species only. *S. krugeri* is noted here for the first time for the territory of Thailand.

## Supplementary Material

XML Treatment for Steatococcus samaraius

XML Treatment for Antonina parazonata

XML Treatment for Antonina vietnamensis

XML Treatment for Geococcus satellitum

XML Treatment for Mollicoccus guadalcanalanus

XML Treatment for Acanthococcus prope onukii

XML Treatment for Gossypariella siamensis

XML Treatment for Coccus viridis

XML Treatment for Discochiton expansum

XML Treatment for Drepanococcus chiton

XML Treatment for Luzulaspis australis

XML Treatment for Megalocryptes buteae

XML Treatment for Megapulvinaria maxima

XML Treatment for Saccharolecanium krugeri
